# Hyperprolactinaemia

**DOI:** 10.3390/jcm8122203

**Published:** 2019-12-13

**Authors:** Irene Samperi, Kirstie Lithgow, Niki Karavitaki

**Affiliations:** 1 Institute of Metabolism and Systems Research, College of Medical and Dental Sciences, University of Birmingham, Birmingham B15 2TT, UK; Irene.samperi89@gmail.com (I.S.); Kirstie.Lithgow@albertahealthservices.ca (K.L.); 2 Centre for Endocrinology, Diabetes and Metabolism, Birmingham Health Partners, Birmingham B15 2TH, UK; 3 Department of Endocrinology, Queen Elizabeth Hospital, University Hospitals Birmingham NHS Foundation Trust, Birmingham B15 2TH, UK

**Keywords:** prolactin, hyperprolactinaemia, prolactinoma, antipsychotics, hypogonadism, dopamine agonists

## Abstract

Hyperprolactinaemia is one of the most common problems in clinical endocrinology. It relates with various aetiologies (physiological, pharmacological, pathological), the clarification of which requires careful history taking and clinical assessment. Analytical issues (presence of macroprolactin or of the hook effect) need to be taken into account when interpreting the prolactin values. Medications and sellar/parasellar masses (prolactin secreting or acting through “stalk effect”) are the most common causes of pathological hyperprolactinaemia. Hypogonadism and galactorrhoea are well-recognized manifestations of prolactin excess, although its implications on bone health, metabolism and immune system are also expanding. Treatment mainly aims at restoration and maintenance of normal gonadal function/fertility, and prevention of osteoporosis; further specific management strategies depend on the underlying cause. In this review, we provide an update on the diagnostic and management approaches for the patient with hyperprolactinaemia and on the current data looking at the impact of high prolactin on metabolism, cardiovascular and immune systems.

## 1. Introduction

Prolactin (PRL) was first discovered between the late 1920s and early 1930s after different authors independently demonstrated lobular-alveolar development and lactation in rabbits after injection of an anterior pituitary extracts [1,2]. In 1933, the “pro-lactin” hormone was purified from animals and was given its name by Riddle et al. [3]. It was first separated from growth hormone (GH) in human pituitary glands by Friesen et al. in 1970 [4], as prior to this, radioimmunoassay differentiating PRL from GH was not available [1,2,5,6,7]. Though initially recognized for its lactogenic action, PRL is now understood to act at many different tissues producing a diverse range of biological functions [8]. PRL belongs to the PRL/GH/placental lactogen family, the members of which share common structural, functional, and binding properties, and arise from a common ancestral gene [2,8]. 

Secretion of PRL by the pituitary gland has a circadian rhythm with higher levels during sleep and lower during wakefulness [6]. Increased concentrations are also seen during ovulation [9]. Synthesis and secretion of PRL is regulated through both inhibiting and releasing factors [8]. PRL inhibiting factors include dopamine, gamma-aminobutyric acid (GABA), and somatostatin [8]. Dopamine is the main inhibitory factor involved in PRL regulation and acts through binding to D2 and D4 receptors in the pituitary lactotrophs. This results in down-regulation of PRL gene expression, reduced secretion of PRL, and decreased lactotroph proliferation [10]. Several hormones and neuropeptides have been suggested to promote PRL release including thyrotropin-releasing hormone (TRH), endogenous opioids, oxytocin, serotonin, vasopressin, vasoactive intestinal polypeptide (VIP), neurotensin, galanin, and salsolinol [8,11]. Nonetheless, PRL is unique from other pituitary hormones in that a physiological “PRL-releasing factor” has not been as yet identified [11]. PRL is also produced in extra-pituitary sites including the ovaries, mammary glands and endometrium, prostate, lymphocytes and hematopoietic cells, skin, adipose tissue, thymus and lymphatic system, endothelium, and the brain [12,13]. It is structurally identical to pituitary PRL and binds to the same receptor [13]. Its regulation is site specific and differs from that of pituitary PRL [13]. 

The PRL receptor (PRL-R) is a single membrane-bound protein of the cytokine receptor superfamily and is structurally and functionally similar to the GH receptor [8]. It is expressed in the pituitary and in many other tissues including the mammary gland, endometrium, ovaries, heart, lung, thymus, spleen, liver, pancreas, kidney, adrenal gland, skeletal muscle, brain, skin and osteoblasts [8,13]. 

The lactotrophic and reproductive actions of PRL are well established, and in recent years, there has been enhanced understanding of the many other biological effects of extra pituitary PRL in humans [9,13]. In women, it is involved in follicular development and maintenance of the corpus luteum, and also acts on the mammary gland to induce and maintain lactation [14]. In the adrenals, it stimulates the secretion of androgens [including dehydroepiandrosterone (DHEA)], cortisol, and aldosterone [9]. PRL has been implicated in the stress-induced activation of the hypothalamic-pituitary-adrenal (HPA) axis [9,13,15]. It increases secretion of ACTH, induces adrenal hypertrophy and storage of cholesterol esters, and stimulates cortisol production [9,15]. PRL produced from lymphocytes and hematopoietic cells has been postulated to be involved in the immune response to stress [8,9,13,15]; its action appears to be dose-related, with immune stimulation at modest and inhibition at high levels [9]. It has varying effects on bone during development and reproduction; in the foetus, it promotes bone growth and mineralization, whereas during pregnancy, it contributes to the accelerated bone resorption providing micronutrients to the foetus [16]. It has vasoconstrictive action and a role in hypertension and pre-eclampsia has been postulated [14]. Finally, it is thought to be involved in osmoregulation by increasing water and salt absorption in the bowel and reducing renal Na+ and K+ excretion [9].

Further possible actions of extra-pituitary PRL have been explored in animal studies. Knock-out PRL-R male mice have infertility proposing a role of PRL in spermatogenesis [10]. Animal models have also suggested metabolic actions including stimulating insulin release, beta-cell proliferation, and adipogenesis [10], whereas in the central nervous system, PRL promotes remyelination [9,17].

## 2. Causes and Mechanisms of Hyperprolactinaemia

The aetiology of hyperprolactinaemia is broad and can be divided into physiological, pathological, and pharmacological causes (Table 1) acting though a number of different mechanisms [6]. 

### 2.1. Physiological Causes

#### 2.1.1. Endogenous Oestrogens

Oestrogens enhance PRL secretion via different proposed mechanisms including regulation of PRL gene expression, downregulation of dopamine receptor expression, and stimulation of lactotroph cell hyperplasia [8,11]. Accordingly, PRL levels are influenced by the menstrual cycle, menopause, and pregnancy, and any high oestrogen state (e.g., pregnancy) is a potential cause hyperprolactinaemia [18,19]. Tanner et al. assessed PRL concentrations in 6540 subjects including men, non-pregnant women at different phases of the menstrual cycle, and post-menopausal women [18]. Pre-menopausal women had significantly higher values compared with post-menopausal ones and men [18]. Furthermore, PRL was significantly higher during ovulation, leading the authors to advocate for the use of specific reference intervals according to the phase of menstrual cycle, or measurement of PRL in the early follicular phase [18]. 

Hyperprolactinaemia is a well-established finding during normal pregnancy [19]. In a study of 994 women, non-pregnant, pregnant, or post-partum, Hu et al. [19] found PRL levels progressively increasing across the three trimesters of pregnancy; 29.19 to 168.44 µg/L for the first trimester, 67.3 to 251.43 µg/L for the second, and 192.1 to 457.48 µg/L for the third one. 

Increases in PRL can occur with use of oral contraceptives containing high doses of oestrogen (35 mcg) [20] but this has not been shown with modern contraceptives with lower amounts of oestrogen [19,21]. 

#### 2.1.2. Breastfeeding

Suckling during nursing is a strong physiologic stimulus for PRL secretion [8] possibly by releasing the lactotrophs from the tonic inhibition of dopamine [8,22]. TRH, vasopressin, oxytocin, and salsolinol have also been proposed as factors involved in the suckling-induced PRL release [8,22]. 

#### 2.1.3. Stress

Hyperprolactinaemia with values usually up to 100 µg/L may be observed as a response to stress [6,15,23] but the responsible mechanism has not been fully elucidated. It has been postulated that stress-induced changes in dopamine and serotonin may affect PRL release contributing to hyperprolactinaemia in this setting [15].

#### 2.1.4. Exercise

Exercise increases PRL in a magnitude proportional to the intensity and duration of the activity [24,25]. Elevation in PRL has been observed following both aerobic and anaerobic exercise but it is greatest in high-intensity anaerobic exercise such as interval training [24]. The peak PRL is thought to occur following exercise [24], and, therefore, it is suggested to avoid vigorous exercise for at least 30 min before blood sampling for PRL measurement [26]. The mechanism of exercise induced-hyperprolactinaemia has not been not fully elucidated. 

#### 2.1.5. Chest Wall Injury

Different processes causing chest wall irritation such as burns, herpes zoster, or injury have been associated with hyperprolactinaemia [25]. This is thought to take place via a neural mechanism interfering with dopamine transmission, as that occurs with suckling [8,22]. Despite the finding that lactation and nipple stimulation can increase PRL [8], breast examination, breast ultrasound, or mammography do not appear to cause hyperprolactinaemia [27,28,29].

### 2.2. Pathological Causes

#### 2.2.1. Prolactin-Secreting Pituitary Tumours 

The most common pituitary cause of hyperprolactinaemia is the presence of prolactinoma. This is the most frequent subtype of pituitary adenoma (accounting for 57% of all pituitary adenomas), and presents more commonly in females with a peak prevalence in women aged 16 to 48 years [30]. 

Prolactinomas are classified by their size, with microprolactinomas measuring <1 cm and macroprolactinomas ≥1 cm (giant ones measure >4 cm) [25,31]. In general, the degree of hyperprolactinaemia correlates with prolactinoma size, however, there are some important caveats to this which will be addressed in the Diagnosis section below. For microprolactinomas, PRL is usually 100 to 200 µg/L, however, outliers exist; in the Brazilian Multicenter Study on Hyperprolactinaemia which included 444 patients with microprolactinoma, mean PRL was 165 µg/L but ranged from 32 to 525 µg/L [32]. Hyperprolactinaemia is also seen in adenomas co-secreting GH and PRL [33,34].

Based on the 2017 WHO classification of pituitary adenomas, morphological variants of the lactotroph adenomas are the sparsely granulated, the densely granulated, and the acidophil stem cell adenoma which also expresses GH [35]. PRL expression is also seen by immunohistochemistry in pluri-hormonal Pit-1 adenomas, mammosomatotroph adenomas, and mixed somatotroph-lactotroph adenomas [35].

Prolactin secreting adenomas can be part of genetic syndromes, such as multiple endocrine neoplasia type 1 (MEN1) or type 4 (MEN4), familial isolated pituitary adenoma (FIPA) or Carney complex [36]. MEN1 is an autosomal dominant condition usually due to loss of heterozygosity of the *MEN1* gene which encodes the menin protein [36]. Clinical features of MEN1 include primary hyperparathyroidism, pituitary adenomas, and pancreatic neuroendocrine tumours [36,37]. Although less than 3% of pituitary adenomas are associated with MEN1, they are present in 30–40% patients with this syndrome [36,37] with prolactinoma being the most common subtype [36,37,38]. Inactivating germline mutations of the aryl hydrocarbon receptor interacting protein (AIP) gene are found in 20% of families with FIPA [39]. Although *AIP* mutations are usually associated with GH-secreting adenomas, they can also be detected in patients with prolactinoma [39]. Carney complex is an autosomal dominant condition characterized by spotty skin pigmentation, myxomas, and multiple endocrine hyperfunction; it is most commonly caused by an inactivating germline mutation in the *PRKAR1A* gene and the protein it encodes is a type 1A regulatory subunit of protein kinase A [36]. Pituitary adenomas occur in 10 to 20% of patients with Carney complex, most are GH secreting tumours but some of them co-secrete GH and PRL [36,40].

#### 2.2.2. “Stalk-Effect”

Any lesion leading to interruption of the dopaminergic pathways due to compression of the pituitary stalk and portal vessels can lead to hyperprolactinaemia through the “stalk effect” [including pituitary adenomas with the most common cause in this setting being the non-functioning pituitary adenomas (NFA); parasellar tumours (such as Rathke’s cleft cyst, craniopharyngioma, meningioma or germinoma); metastatic pituitary disease, infiltrative/inflammatory diseases (such as lymphocytic hypophysitis, Langerhans cell histiocytosis, and sarcoidosis)] [6,25,41]. Stalk compression from NFA or other parasellar tumours is typically associated with PRL levels <100 µg/L [42,43]. Hyperprolactinaemia has also been reported in 7 to 10% of patients with primary empty sella syndrome attributed to compression of the pituitary gland against the sellar walls causing stretching of the pituitary stalk [44]. 

#### 2.2.3. Renal Failure

Hyperprolactinaemia is common in patients with renal disease and is recognized as a cause of hypogonadism and sexual dysfunction in these patients [25,45]. PRL usually ranges between 30 and 108 µg/L, although rarely, much higher concentrations exceeding 600 µg/L may be observed [45]. Increased PRL secretion and decreased PRL clearance are both thought to contribute [25,45]. Hyperprolactinaemia resolves following successful renal transplantation [25]. 

#### 2.2.4. Liver Cirrhosis 

Hyperprolactinaemia is common amongst patients with cirrhosis with levels less than 100 µg/L. Its degree correlates with the severity of liver disease [46]. The mechanism is thought to be secondary to decreased dopamine-mediated PRL inhibition, as well as increased circulating oestrogen levels [46].

#### 2.2.5. Primary Hypothyroidism

It has been suggested that approximately 40% of cases of primary hypothyroidism are associated with hyperprolactinaemia [47,48] caused by stimulation of lactotroph cells from the increased TRH levels [9]. Its magnitude correlates with the degree of TSH elevation [47]. Hyperprolactinaemia is typically mild with values <100 µg/L [47,48]. A further mechanism of hyperprolactinaemia secondary to untreated primary hypothyroidism is thyrotroph hyperplasia, which can mimic a pituitary adenoma and induce hyperprolactinaemia via the “stalk effect” [25,49].

#### 2.2.6. Polycystic Ovarian Syndrome

Hyperprolactinaemia has been reported in 7 to 52% of women with polycystic ovarian syndrome (PCOS) [50,51]. Its pathogenesis has not been elucidated; oestrogen-mediated stimulation of lactotrophs and relative dopamine deficiency have been proposed [50,51]. Given the presence of PRL receptors in the adrenal gland [8], it has also been postulated that hyperprolactinaemia stimulates adrenal androgen production contributing to the hyperandrogenism of PCOS [51]. It should be noted that whether hyperprolactinaemia is truly a clinical feature of PCOS remains controversial. A case-control study of 82 women with PCOS showed no difference in the prevalence of hyperprolactinaemia compared to women with insulin resistance, and notably, all cases of hyperprolactinaemia in the women with PCOS were finally attributed to a prolactinoma or medication use [52]. 

#### 2.2.7. Seizures

Hyperprolactinaemia following seizures is transient with peak levels 10 to 20 min post-seizure and resolution within 2 to 6 h [53]. It has been proposed that this occurs due to propagation of epileptic activity from the temporal lobe to the hypothalamo-pituitary axis [54]. It is most common following generalized tonic-clonic seizures and can also be seen after alcohol withdrawal seizures or complex partial seizures; it is rare following simple partial or psychogenic non-epileptic seizures [53]. PRL is, therefore, a potentially useful biomarker in differentiating epileptic seizures from non-epileptic ones but the sensitivity and specificity of this are highly variable across different series [53]. 

### 2.3. Pharmacological

A number of drugs can alter PRL homeostasis leading to hyperprolactinaemia via different mechanisms. The most common culprit medications are antipsychotics/neuroleptics and antidepressants [6,55] and the main mechanisms involve reduction in dopamine transmission and increase in serotonin transmission (with the latter leading to augmentation of PRL releasing factors, like oxytocin and VIP) [55,56]. 

The prevalence of hyperprolactinaemia amongst different classes of medications is shown in Table 2.

#### 2.3.1. Antipsychotics

Antipsychotics are typically divided into those with PRL-rising and PRL-sparing potential [56]. Possible explanations for these differences include variations in the speed of dissociation from the D2R, ability to cross the blood-brain barrier, presence of D2R polymorphisms, and degree of serotonergic inhibition [55,56,59]. First generation antipsychotics are typically associated with more severe hyperprolactinaemia (two to three times the upper limit of normal) [55]. Second generation antipsychotics have lower D2R affinity and stronger 5HT2A receptor blockage leading to milder PRL elevations [55]. Exceptions to this are risperidone, paliperidone and amisulpride, all second-generation antipsychotics that can cause significant hyperprolactinaemia [55,56]. Both oral and depot formulations of risperidone and paliperidone increase PRL in a dose dependent manner. Amisulpride is thought to have the greatest potential for hyperprolactinaemia of all antipsychotics; these effects occur independent of dose and can resolve rapidly after discontinuation of the drug [55]. Aripiprazole and quetiapine show the lowest possibility for hyperprolactinaemia [55,56]. 

The PRL raising potential of commonly used first and second-generation antipsychotics is shown in Table 3. 

#### 2.3.2. Antidepressants 

Different classes of antidepressants, including monoamine oxidase inhibitors, tricyclic antidepressants, and selective serotonin reuptake inhibitors (SSRIs) induce hyperprolactinaemia mainly via serotonergic pathways [55,59]. This is typically mild and it is rarely symptomatic [59]. 

#### 2.3.3. Antiemetics

The antiemetics domperidone and metoclopramide antagonize D2R and can acutely induce hyperprolactinaemia [55,59]. These effects can be harnessed for increasing breast milk production for breastfeeding [64].

#### 2.3.4. Opioids

Opioids can cause hyperprolactinaemia mediated by μ-, κ- and δ-opioid receptors in the hypothalamus [65]. Acute oral or *iv* opioid administration can induce hyperprolactinaemia in men and post-menopausal women, while the findings after chronic opioid use have been mixed [65]. High PRL has been found in individuals with opioid addiction, with opium smoking and on oral opioids for chronic pain, but not with intrathecal morphine or opioid maintenance therapy with buprenorphine or methadone [65]. 

#### 2.3.5. Antihypertensives 

Verapamil is a non-dihydropyridine calcium channel blocker of the phenylalkylamine class [66]. This specific class of calcium channels blocker causes hyperprolactinaemia due to suppression of tuberoinfundibular dopamine; this is not seen with the other commonly used non-dihydropyridine agents diltiazem and nifedipine [67]. Methyldopa is a competitive inhibitor of the enzyme DOPA decarboxylase and causes hyperprolactinaemia through inhibition of dopamine synthesis [58]. Reserpine is an alkaloid used for treatment of hypertension and psychosis; it reduces dopamine transmission by blockade of vesicular monoamine transporter type 2 in monoamine neurons [68]. 

## 3. Clinical Manifestations 

The clinical manifestations of hyperprolactinaemia mainly relate with the reproductive system [25,43]. The patients may also have other signs and symptoms depending on the aetiology of the PRL excess (e.g., mass effects, in case of a pituitary tumour (headaches, visual disturbances, hypopituitarism)). 

### 3.1. Reproductive Manifestations

#### 3.1.1. Women

Hyperprolactinaemia suppresses GnRH (via reduction of kisspeptin in the hypothalamic arcuate and periventricular neurons) leading to a decrease in LH pulse amplitude and frequency [43]. It also decreases ovarian oestrogen and progesterone production [43]. These result in menstrual irregularities (amenorrhoea or oligomenorrhoea), infertility, decreased libido and galactorrhoea [25]. Galactorrhoea (spontaneous or provoked) is a common manifestation in pre-menopausal females (up to 90%), but occurs less often in post-menopausal ones as the mammary glands are not primed with oestrogen and progesterone [69]. 

Hyperandrogenism may be also a manifestation of hyperprolactinaemia due to stimulation of adrenal androgen production [51,70,71,72]. In a prospective study including 101 medically and 22 surgically treated patients with prolactinoma and normal cortisol reserve, decreases in the adrenal steroid DHEA-S paralleled PRL reduction after treatment with dopamine agonist or surgery [72]. However, this study did not report on clinical symptoms of hyperandrogenism [72]. It is also unclear whether there is a link between hyperprolactinaemia and female pattern hair loss [73]. It is possible that PRL exacerbates hyperandrogenism in clinically predisposed women, such as those with PCOS [51] but further studies are needed on this topic.

#### 3.1.2. Men 

In males, hyperprolactinaemia causes hypogonadotropic hypogonadism [74] manifesting with reduced libido, erectile dysfunction, and impaired spermatogenesis [75]. Gynaecomastia (secondary to hypogonadism) and galactorrhoea occur rarely [75]. Anaemia, decreased energy and muscle mass may be also present as secondary manifestations of hypogonadism [71,76]. 

### 3.2. Bone Manifestations 

It is well established that hypogonadotropic hypogonadism has negative impacts on bone health [77]. Furthermore, in vitro studies have demonstrated that PRL has variable effects on bone metabolism, depending on its levels. PRL within the physiological range stimulates bone formation, mild hyperprolactinaemia increases bone resorption, and marked hyperprolactinaemia further increases bone resorption and inhibits bone formation [77]. This is supported by findings that patients with prolactinoma have suppressed osteocalcin, high NTX (collagen type I crosslinked N-telopeptide), and increased RANKL (receptor activator of nuclear factor-kB ligand)/OPG (osteoprotegerin) ratio, suggesting increased bone resorption and decreased bone formation [77,78]. The relative contribution of hypogonadotropic hypogonadism and hyperprolactinemia is controversial and it has been hypothesized that these mechanisms have synergistic effects on bone loss [77]. In vitro and animal studies have suggested that hyperprolactinaemia directly stimulates bone resorption independently of hypogonadism [77] but further research on this topic is needed. 

Bone loss affects both men and women with prolactinoma, and reduced bone mineral density is more common in the lumbar spine than in the hip, suggesting that trabecular bone may be damaged earlier than cortical bone [77,79]. In a case control study, higher rates of vertebral fractures were found in women with prolactinoma compared with controls [80]. A systematic review and meta-analysis demonstrated that rates of vertebral fractures were significantly lower in treated *vs.* untreated men and women with prolactinoma, independent of gonadal function [81].

## 4. Diagnostic Approach

### 4.1. Blood Sampling for PRL

Current guidelines state that serum PRL can be drawn at any time of day [25]. Exercise and nipple stimulation should be avoided for at least 30 min prior to testing [26].

Typically, normal PRL levels are <20 µg/L in men and <25 µg/L in women [82], however, it is important to interpret the results according to the assay specific reference ranges [83], and also considering pre/post-menopausal status. [18,19]. A single PRL value above the upper limit of normal can be sufficient for confirmation of hyperprolactinaemia, provided blood sampling took place without excessive venepuncture stress [25]. However, if there is doubt, particularly in cases of mildly elevated PRL, sampling could be repeated at 15 to 20 min intervals to account for pulsatility [9,25]. Dynamic tests are not currently used for establishing the diagnosis of hyperprolactinaemia [25]. 

### 4.2. Biochemical Interpretation 

#### 4.2.1. Macroprolactin

Most circulating PRL (23 kDa) is monomeric, however, serum also contains larger isoforms including dimeric PRL or big PRL (45 to 60 kDa) and polymeric or big-big PRL (150 to 170 kDa) [25,26]. Big-big PRL, termed macroprolactin, is mostly complexes of prolactin-IgG, prolactin-IgA, or polymeric aggregates of highly glycosylated PRL monomers [84]. In most individuals, the majority of serum PRL (~85%) is monomeric but in others there is preponderance of macroprolactin, referred to as macroprolactinaemia [25,26]. The prevalence of macroprolactinaemia amongst cases with hyperprolactinaemia is reported between 4% and 40% [25,26,85]. Although macroprolactin is not considered to have significant biological activity, it retains partial or total immunoreactivity with anti-PRL antibodies used in commercial immunoassays [83,84,85]. Detection of macroprolactin is clinically important to avoid incorrect diagnosis and unnecessary investigations [83]. Gel filtration chromatography is regarded as the “gold standard” technique, however, it is labour-intensive and expensive [83,84,86]. Polyethylene glycol (PEG) precipitation is the most widely used method, as it is simple, fast, and cost effective [25,83,84,86]. It involves mixing and incubating the serum with 25% PEG which is then centrifuged and the precipitating PRL complexes are subsequently quantified [83,84,86]. Recovery of free PRL > 60% of the total is suggestive of monomeric hyperprolactinaemia, whereas recovery <40% is consistent with macroprolactinaemia [33,86]. 

Despite the general view that macroprolactin does not have significant biological activity, in recent years, this has been a matter of debate [83,85]. Thus, it has been proposed that macroprolactin can dissociate into biologically active monomeric isoforms; nonetheless, studies assessing the biological activity of macroprolactin in vitro have yielded conflicting results [83,85,86]. Kalsi et al. [87] in a study of 102 hyperprolactinaemic individuals, found that 68.8% of those with macroprolactinaemia had reproductive manifestations that could not be otherwise explained. This led the authors to propose that macroprolactin may have some degree of biological activity [87] but these findings require further validation. In daily practice, if macroprolactin is not assessed in local hospital laboratories, this can be evaluated in most reference laboratories upon request. 

#### 4.2.2. Hook Effect 

Hook effect is another analytical issue important in clinical practice [25,26]. It is encountered in the presence of excessively high PRL levels (as seen with macro- or giant prolactinomas) which saturate the antibodies used in the two-site immunoradiometric assay leading to artificially low PRL values [25,26]. Failure to recognize this effect may result in missing the diagnosis of a prolactinoma and to incorrect therapeutic decisions [88]. Large pituitary macroadenomas (>3 cm with expected PRL level >10,000 µg/L) with only mild hyperprolactinaemia should raise suspicion for the hook effect, and close interaction between clinicians and biochemists is required in this scenario [25,26]. It can be overcome by repeating the assay on 1:100 serum sample dilutions, or by performing a washout just after the binding of PRL to the first antibody aiming to eliminate the excessive unbound PRL [25,26]. As many newer PRL assays are no longer susceptible to the hook effect, clinicians should consult with their local laboratories when querying this issue [25]. 

### 4.3. Imaging

The diagnostic work-up of hyperprolactinaemia includes imaging of the sellar area, which is performed after excluding other common causes of high prolactin (physiological, primary hypothyroidism, drug-induced). Pituitary MRI (magnetic resonance imaging) enhanced with gadolinium is the gold standard approach [82]. 

### 4.4. Diagnostic Algorithm

In patients on PRL-elevating drugs, PRL should be repeated after withdrawal of medications for 72 hours, if possible [25,56]. However, this approach may not be safe if this treatment is offered for psychiatric indications [89]. If stopping the drug is not feasible, pituitary MRI is advised to rule out a sellar/parasellar tumour [25,56]. Due to the challenges in investigating hyperprolactinaemia in such scenarios, checking baseline PRL levels prior to initiation of antipsychotic medication is recommended aiming to avoid future unnecessary imaging [56].

**Figure 1:**
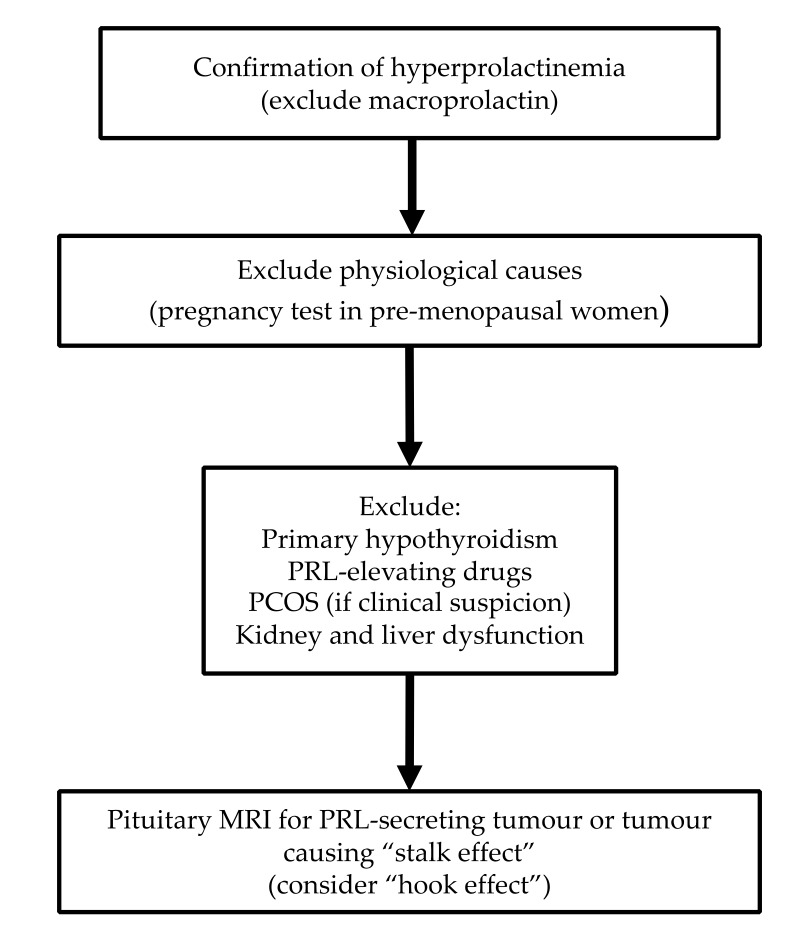
Proposed algorithm for investigating hyperprolactinaemia.

The differentiation between a PRL-secreting tumour and a NFA or other sellar/parasellar mass causing hyperprolactinaemia through “stalk effect” is very important, as these conditions are managed differently [42]. In prolactinomas, apart from the cystic ones [43], adenoma size generally corresponds with PRL levels. Nonetheless, there is no PRL cut-off with perfect diagnostic value differentiating prolactinoma from other tumours [42]. Karavitaki et al. [42] in a series of 226 histologically proven NFAs showed that 99% of them had serum PRL of <2000 mU/L (~100 µg/L) suggesting that PRL above this limit is most likely consistent with a prolactinoma. Rarely, long-standing untreated primary hypothyroidism can cause significant lactotroph hyperplasia which may mimic a pituitary adenoma [49]. A proposed diagnostic algorithm for hyperprolactinaemia is shown in Figure 1. 

## 5. Management

The main goals of therapy for hyperprolactinaemia are restoration and maintenance of normal gonadal function/fertility, and prevention of osteoporosis [74]. Specific management strategies depend on the underlying aetiology [25,43]. 

### 5.1. Prolactinomas 

The therapeutic targets in prolactinomas are to normalize PRL, reduce tumour size, resolve clinical manifestations of hyperprolactinaemia and of mass effects (particularly visual disturbances) and prevent progression or recurrence of the tumour [25,74]. 

#### 5.1.1. Conservative Management

Based on current guidelines, patients with microprolactinoma who are asymptomatic or post-menopausal do not require treatment [25,82,90]. However, some authors advocate treatment in post-menopausal women due to the postulated negative effects of hyperprolactinaemia on bone health, weight gain, and insulin resistance [77,91]. Post-menopausal women managed with a conservative strategy require ongoing surveillance for tumour progression [25,82,90]. 

#### 5.1.2. Dopamine Agonists

Dopamine agonists (DA) are first line treatment for patients with prolactinoma [25,43,82]. They induce apoptosis, autophagic cell death and paraptosis of lactotrophs through different molecular pathways mediated by D2R, leading to reduced prolactin secretion and tumour shrinkage [92]. Available DAs include bromocriptine, cabergoline, and quinagolide [25,43,82]. Bromocriptine is a semisynthetic ergot derivative with D2R agonist activity and D1R antagonist activity requiring daily administration due to its short half-life; starting doses are 0.625 to 1.25 mg/day and can be titrated to 2.5 to 15 mg/day [25,82,93]. Cabergoline is a D2R selective agonist with longer half-life allowing a single to twice weekly administration; typical starting doses are 0.25 to 0.5 mg once or twice weekly with titration to usual doses of 0.5 to 3 mg weekly. [25,82,93]. Quinagolide is a non-ergot derived D2R agonist with a 22-hour half-life which allows for daily dosing; typical starting doses are 25 mcg daily usually titrated to 75-150 mcg daily [93]. Current guidelines recommend cabergoline to be used as first line over bromocriptine due to its superior efficacy (attributed to greater affinity and selectivity for D2R) and more optimal side effect profile [25,43,82,92]. Improved adherence due to fewer side effects and more convenient dosing schedule are also further advantages [25,92]. Results from cumulative studies have demonstrated that DAs result in reduction in tumour size in 20 to 100% (median 62%), resolution of visual field defects in 33 to 100% (median 67%), resolution of amenorrhea in 40 to 100% (median 78%), resolution of infertility in 10 to 100% (median 53%), improvement of sexual function in 6 to 100% (median 57%), resolution of galactorrhoea in 33 to 100% (median 86%), and normalization of PRL in 40 to 100% (median 68%) of the patients [25]. 

Common side effects of DAs include nausea and/or vomiting and postural hypotension which can lead to dizziness and syncope, a digital Raynaud-type syndrome, and, more rarely, leg cramps, flushing, and nasal congestion [94]. High dose DA (as offered in Parkinson’s syndrome) increases the risk of cardiac valvulopathy [95] but the risk of clinically significant valvulopathy in patients with prolactinoma is still not clearly established [96]. Most studies on prolactinoma patients do not show an elevated risk [97,98,99] but, given the uncertainty around the risk of valvulopathy in this setting, current guidelines recommend screening echocardiography prior to initiating DA, and thereafter every five years for patients taking cabergoline less than 2 mg per week or annually for those on more than 2 mg per week [100].

Rarely, DAs can induce mood changes and psychosis in susceptible patients [101]. More subtle psychiatric symptoms manifesting as impulse control disorders are also increasingly being recognized [102]. Pituitary apoplexy has been reported during use of DA in a small number of cases [103] and usually this occurs in patients with macroprolactinoma during the first 1.5 years of treatment [103,104,105]. 

Guidelines suggest that withdrawal of DA may be attempted after at least two years of therapy if PRL has normalized and there is no visible tumour on MRI; remission rates of 26 to 69% have been observed following discontinuation [25,82]. Withdrawal of DA carries risk of tumour recurrence or regrowth, particularly in the first year, and ongoing regular surveillance is required [25,82]. DA withdrawal may also be considered in women once they become post-menopausal [25], however, prolactinoma regrowth has been observed in this setting, and continued surveillance is required [90].

In case of pregnancy, DA should be discontinued, except in selected patients with macroadenoma at risk of visual deterioration [25]. Although there is longer experience with use of bromocriptine than with cabergoline during pregnancy, both have demonstrated safety in this setting [106]. The risk of prolactinoma growth during pregnancy is <2% for microprolactinomas and around 18% for macroprolactinomas [25,43,82,106]. If visual deterioration occurs, management options include reinstitution of DA or surgical debulking [25]. If surgery is to be undertaken, the preferred timing is during the second trimester [43]. If the pregnancy is at or near term, induction of delivery may also be considered [25,43]. Remission rates following pregnancy of 10 to 60% (median 27%) have been reported, and breastfeeding does not appear to increase the risk of tumour growth [43,107,108]. 

Normalization of PRL and/or tumour shrinkage cannot be achieved with DA treatment in all cases of prolactinomas [25,109]. Prolactinomas are labelled as DA resistant due to either failure to achieve normoprolactinaemia and/or failure to achieve 50% reduction in tumour size with maximum tolerated DA dose (can be up to 30 mg daily for bromocriptine or 12 mg weekly for cabergoline [110,111]) after at least 6 months of treatment [109]. DA resistance occurs in approximately 20 to 30% of patients on bromocriptine and around 10% of patients on cabergoline [109]. Male sex, younger age at diagnosis, tumour invasiveness (particularly in the cavernous sinus) and size >4 cm have been proposed as clinical factors associated with DA resistance [109,112]. In such cases, switching bromocriptine to cabergoline is an option, leading to normalization of PRL in 80 to 85% of patients [109]. On the other hand, changing cabergoline to bromocriptine is rarely effective [111] but systematic data are not available. For patients already on cabergoline, dose escalation is another option, as a number of them (around 15 to 20%) may require higher than the conventional doses of 2 mg per week [25,111]. It is uncommon for a prolactinoma initially responsive to DA to become resistant to this treatment; if this occurs, adherence to the DA should be confirmed [43,109,111]. Transsphenoidal surgery and radiotherapy are second and third line management options, respectively, for resistant prolactinomas, as discussed below [25]. 

The above strategies are not always successful in overcoming resistance to DA, and, therefore, alternative medical therapies have been explored [109]. Somatostatin analogues (SSA) alone or in combination with DA have been proposed as a potential approach, due to expression of all somatostatin receptor (SSTR) subtypes in prolactinomas [109]. However, results from small case series using first generation SSA (octreotide and lanreotide) have been conflicting [109]. Second generation SSA (pasireotide) may confer a theoretical advantage over first generation ones due to greater affinity for SSTR5, however, successful experience with this agent is limited to two case reports [109]. Due to the effect of oestrogens on lactotroph proliferation and hyperplasia, case reports and small case series have explored use of selective oestrogen receptor modulators and aromatase inhibitors for DA resistant prolactinomas, but the results of these studies are not conclusive [109]. Metformin, tyrosine kinase inhibitors (EGFR and VEGF inhibitors), and mTOR inhibitors have attracted interest, but clinical evidence is limited to case reports, necessitating further work on this field [109]. 

Temozolomide (TMZ) has been used as first line chemotherapeutic agent in lactotroph carcinomas and aggressive prolactinomas [109]. Lower expression of the DNA repair protein O6-methylguanine-DNA methyltransferase (MGMT) has been proposed to correlate with TMZ effectiveness and checking for the MGMT tumour status by immunohistochemistry prior starting treatment is recommended [109]. Response to TMZ within three months of treatment is a predictor of efficacy; discontinuation is advised if there is radiological progression after that interval [109]. 

#### 5.1.3. Surgery

Surgery could be considered for prolactinomas where maximum doses of DA are not effective or tolerated, for those presenting with pituitary apoplexy with visual deterioration, when there are cystic tumour components compressing the visual pathway, or if cerebrospinal fluid leak develops [25,43,110,111,113]. Surgery may also be an option for young patients with microprolactinoma, as this may be a more cost-effective approach compared with lifelong DA therapy [114]. Rates of post-operative remission are widely variable across the literature, and are reported 38 to 100% for microprolactinomas and 7 to 80 % for macroprolactinomas [115]; lower rates of remission are described in tumours with cavernous sinus invasion [111,116]. Higher success rates are seen in the hands of experienced pituitary surgeons [114]. DA resistant prolactinomas may show improved responsiveness to these agents following debulking surgery [110,111]. 

#### 5.1.4. Radiotherapy 

Radiotherapy is a third line option for prolactinomas that have failed medical and surgical management, or for aggressive or malignant ones [25,43,110]. Although it can be effective in controlling tumour growth, normalization of PRL is reported in only one third of patients, and it may require up to 20 years for maximal effects to be seen [25,43,111]. Pituitary irradiation is associated with late adverse effects mainly including hypopituitarism, optic nerve damage, cranial nerve dysfunction, as well as increased risk of stroke and second brain tumours [25,82,117].

### 5.2. “Stalk Effect”

Sellar/parasellar tumours leading to hyperprolactinaemia due to the “stalk effect” are usually managed surgically [118]. In a series of 267 patients with NFA, 72 had hyperprolactinaemia secondary to “stalk effect” and eight of them had galactorrhoea [119]. At 12 weeks postoperatively, PRL normalized in 92% and all cases of galactorrhoea resolved [119]. In selected cases, there may be clinically significant hyperprolactinaemia from the “stalk effect” in the absence of other indications for surgery, such as hormone hypersecretion and visual field compromise. In these patients, hypogonadism should be treated, and if galactorrhoea is also bothersome, a pragmatic approach is to trial DA [120]. Notably, DA treatment has been associated with reduction in the rate of residual adenoma enlargement in patients with NFA who had surgical removal of the tumour [121]. 

### 5.3. Other Pathological Causes

Hyperprolactinaemia secondary to primary hypothyroidism should be managed through treatment of the underlying hypothyroidism with thyroid hormone replacement [25,43]. 

Hyperprolactinaemia secondary to renal failure has been shown to normalize following successful renal transplantation [25]. The use of PRL lowering treatment in patients with hyperprolactinaemia secondary to chronic kidney or liver disease has not been investigated. 

### 5.4. Pharmacological

In hyperprolactinaemia secondary to medication use, the preferred approach is to discontinue the offending drug [25,43]. However, this strategy may not be safe for patients with psychiatric disorders on antipsychotics [89]. Although antipsychotic-induced hyperprolactinaemia is common, there is no consensus on its management due to lack of high quality evidence [89]. Grigg et al. [89] in a systematic review and critical analysis of published guidelines and literature on this topic proposed that, if possible, baseline PRL level should be drawn prior to initiating or changing antipsychotic therapy [89]. Patients with asymptomatic hyperprolactinaemia due to antipsychotics should undergo periodic monitoring of PRL, as well as regular clinical assessments for relevant manifestations [56,89]. For those with symptomatic hyperprolactinaemia, a trial of stopping the antipsychotic medication is not recommended due to risk of exacerbation or relapse of psychiatric symptoms [89]. One option is to decrease the dose of the antipsychotic but this may result in reduced control of psychiatric symptoms, and may also be ineffective, as hyperprolactinaemia in this setting is not always dose related [55,89]. Due to partial agonism at D2R, addition of aripiprazole as an adjunctive therapy has shown to be an effective strategy in reducing hyperprolactinaemia, however, there is concern that this could lead to a reduction in the efficacy of the primary antipsychotic or to increased side effects due to polypharmacy [55,59,89]. A further option is initiating hormone replacement therapy with oestrogen (oral contraceptive pill or hormone replacement therapy depending on whether contraception is required) or testosterone, as appropriate [89]. Switching antipsychotics to reduce hyperprolactinaemia is a last resort and should be undertaken with caution only when the psychiatric condition has been stabilized [89]. Use of DA in this patient population is not currently recommended due risk of worsening psychotic symptoms and lack of established safety [89]. Management of antipsychotics-induced hyperprolactinaemia should be undertaken in close collaboration with psychiatry and be individualized based on the potential benefits and risks of each strategy [89]. 

## 6. Hyperprolactinaemia and Metabolic/Cardiovascular Risk

### 6.1. Metabolic Risk

Animal studies have demonstrated that PRL is implicated in appetite regulation, body weight, and fat deposition [122]. PRL is involved in the development of pancreatic β-cells and has also been postulated to have functional importance in the adult pancreas [123]. It may contribute to insulin resistance and hyperglycaemia through decreasing glucose transporter 4 (GLUT4) expression and inducing pyruvate dehydrogenase kinase 4, thereby, reducing glucose uptake and oxidation [123]. PRL signalling appears to modulate adipocyte differentiation and impact energy homeostasis and thermogenesis [123]. 

In large population-based studies from China and USA, normal to high-normal PRL levels (but within the reference range) were related to favourable glucose metabolism profile and low risk of diabetes mellitus type 2 [124,125]. There is limited data on hyperprolactinaemia and metabolic parameters. PRL may impact insulin resistance; patients with hyperprolactinaemia have higher insulin, HOMA-IR, and HOMA-β index levels compared with healthy controls [126]. A correlation between hyperprolactinaemia and BMI (body mass index) has also been observed in prolactinoma patients independent of hypogonadism [127].

Several studies have evaluated the effect of treatment of hyperprolactinaemia with DA on numerous metabolic parameters. DAs reduce triglycerides, fasting glucose, and LDL cholesterol in individuals with prolactinomas [127]. In contrast, in another study on prolactinoma patients offered DA demonstrated no change in fasting glucose, although significant decrease of LDL and total cholesterol were observed [128]. A report of 20 premenopausal women with prolactinoma described significant decreases in BMI, total cholesterol, and LDL following treatment with DA [129]. Furthermore, in a series of 61 patients with prolactinoma, significant improvements in lipid profile, insulin resistance, visceral adiposity, and prevalence of metabolic syndrome following treatment with cabergoline were found [130]. However, it is difficult to discern whether these favourable metabolic effects are directly related to resolution of hyperprolactinaemia or are due to restoration of gonadal function or a combination of both. Notably, a study of 32 men with prolactinoma demonstrated significant decreases in the prevalence of metabolic syndrome after 12 months of treatment with cabergoline only amongst those with hypogonadism at baseline [131]. Nonetheless, significant improvements in weight, BMI, waist circumference, lipid profile, and insulin resistance were seen in both hypogonadal and eugonadal patients, suggesting that PRL may play a direct role in mediating these adverse metabolic effects [131]. 

### 6.2. Cardiovascular Risk

It has been proposed that hyperprolactinaemia has cardiovascular actions, either direct or indirectly through hypogonadism [132,133]. PRL increases sympathetic tone and mediates chronotropic and vasoconstrictive effects; for this reason, it has been implicated in cardiovascular (CV) risk [132]. In women, PRL >8 µg/L was independently associated with risk of incident hypertension [134]. However, the Framingham Heart Study did not find any association between CV risk and variation in PRL levels (when within the reference range) [135]. Furthermore, differences in PRL values have not been shown between women with different levels of CV risk [132].

Higher concentrations of several cardiac risk markers including total cholesterol, LDL, apo B, platelet count, fibrinogen, AT-III, PAI-1 and PAI-1/t-PA have been reported in patients with prolactinoma [136]. In a retrospective cohort study including 2233 individuals with prolactinoma and 10,355 matched controls, only males were at increased risk of incident cardiovascular disease (incidence rate ratio 1.94; 95% CI: 1.29–2.91, *p* = 0.001) [137]. However, the contribution of possible growth hormone deficiency and/or hypogonadism in these findings cannot be excluded. 

## 7. Hyperprolactinaemia and Autoimmunity

PRL modulates humoral and cellular immune responses through different but not completely understood mechanisms. It induces production of IL-1, IL-6, and interferon γ, and expression of IL-2 receptors, regulates the maturation of CD4+ and CD8+ T-cell, modulates cytokine production, stimulates pro-B cell generation, and reduces the apoptosis of transitional B cells [138,139]. Hyperprolactinaemia is associated with enhanced activation of anergic B cells that promote autoreactivity [138,139]. Moreover, pro-inflammatory cytokines stimulate pituitary PRL secretion [140] and lymphocytes express dopamine receptors (predominantly D4R and D5R) modulating local PRL production and secretion [8]. A recent case control study by Liu et al. [141] of 202 patients with hyperprolactinaemia found that a significantly higher proportion of hyperprolactinaemic patients had at least one detectable autoantibody, as well as higher levels of IL-4 and IL-6 compared to healthy controls. 

### 7.1. Lupus

A systematic review and meta-analysis of 12 studies by Wang et al. demonstrated significantly higher PRL levels in patients with systematic lupus erythematosus (SLE) [142]. It should be noted though that an association between hyperprolactinaemia and SLE has not been confirmed in all reports [143]; in the systematic review by Wang et al. [142], differences in PRL levels between patients and controls were statistically significant in populations from Asia and Europe but not from America. 

Animal studies have found enhanced expression of PRL-R on T-cells, and that metoclopramide-related hyperprolactinaemia induced features of SLE [144,145]. Human studies have also demonstrated an association between hyperprolactinaemia and manifestations of lupus; hyperprolactinaemia in patients with SLE has been associated with higher risk of serositis and anaemia [139], as well as with neurological manifestations, renal involvement, haematological manifestations, and serological disease markers, namely complement and anti-dsDNA [146]. 

### 7.2. Rheumatoid Arthritis

Studies on PRL levels in RA have provided inconsistent results with lower, higher, or equivocal values [139,147]. It has been shown that the risk and severity of RA are decreased in reproductive states characterized by hyperprolactinaemia (pregnancy and breastfeeding) [148]. However, others have demonstrated that severe disease is associated with longer period of breast-feeding and larger number of breastfed children [149]. A randomized placebo controlled study of bromocriptine offered in 88 patients with active RA on background treatment with methotrexate did not have any significant impact on disease activity score at three months [150]. 

Various hypotheses have been proposed for the inconsistent findings. It has been postulated that lower PRL levels are immunostimulatory, whereas higher ones are immunosuppressive [148]. Others have suggested that extra-pituitary PRL is more important in the pathogenesis of RA than pituitary PRL; PRL-receptors have been demonstrated in synovial macrophages, and PRL mRNA expression in synovial tissue is positively correlated with clinical disease activity [147,151]. Further reports have also implicated the significance of extra-pituitary prolactin promoter polymorphisms [152]. 

### 7.3. Multiple Sclerosis 

PRL has been implicated in the pathogenesis of multiple sclerosis (MS) and other demyelinating disorders [149,153]. PRL is thought to have dual effect in the central nervous system; it stimulates regeneration of neurons, oligodendrocytes, and neural stem cells, and also promotes aberrant immune responses through stimulating B cell autoreactivity [149]. The latter mechanism possibly explains the correlation between hyperprolactinaemia and MS [140,149]. Patients with MS have higher PRL levels compared to controls, and this has also been shown in patients with neuromyelitis optica (NMO) [140,153]. Notably, PRL is higher in patients with MS or NMO during attacks compared with phases of remission [153] but it is still unclear whether hyperprolactinaemia is a primary cause or a secondary effect of the disease. 

### 7.4. Other Autoimmune Disorders 

Hyperprolactinaemia has been described in numerous other autoimmune disorders including systemic sclerosis, Behçet’s disease, and polymyositis [138]. In coeliac disease, a positive correlation between serum PRL with disease activity, mucosal atrophy, and concentration of anti-endomysial antibodies has been shown [149]. A small study demonstrated higher levels of the PRL-R in patients with alopecia areata compared to controls, and levels of the PRL-R were correlated with disease severity [154]. Patients with pemphigus vulgaris have also been shown to have higher PRL compared with controls [155]. The pathophysiological significance of these findings remains to be elucidated. 

## 8. Cancer

An association with PRL levels and breast [156,157,158], ovarian [159], colon [160,161] and hepatocellular [162] cancer has been suggested. Although basic science studies have implicated a role for PRL and its receptor in the pathogenesis of different malignancies [157,158,163,164,165,166], a clear causal relationship between PRL and cancer remains controversial [158]. Epidemiological studies on this topic have shown conflicting results [158], and reports suggesting positive association between PRL values and cancer risk should be interpreted with caution, as some include individuals with normal PRL values, or rely on a single measurement of the hormone [156,157,158]. Notably, a study of 1342 women with hyperprolactinaemia treated with DA did not demonstrate increased risk of breast cancer compared with the general population [167]. Well-designed epidemiological and clinical studies are required to investigate reliably this issue. 

## 9. Conclusions

Hyperprolactinaemia is a biochemical diagnosis with a broad range of aetiologies. Detailed history and clinical assessment are important first steps for the differential diagnosis and for verification of pathological causes. Exclusion of macroprolactin and consideration of the “hook effect” are important points in the diagnostic approach. Medications and sellar/parasellar masses (prolactin secreting or acting through the “stalk effect”) are the most common causes of pathological hyperprolactinaemia. 

Treatment of hyperprolactinaemia mainly aims at restoration and maintenance of normal gonadal function/fertility, and prevention of osteoporosis. Specific management strategies depend on the underlying cause. A particularly challenging scenario is drug-induced hyperprolactinaemia, especially when this occurs in association with antipsychotics or other psychiatric medications, as the degree of PRL elevation can be variable and discontinuing or changing the culprit medication may not be safe. DA are the mainstay of therapy for prolactinomas and specific guidelines are available in clinical practice. 

Recent data expand the biological effects of prolactin particularly on metabolism, cancer, cardiovascular and immune systems opening new avenues on the clinical implications of prolactin and the consequences of hyperprolactinaemia.

## Figures and Tables

**Table 1:** Main Causes of Hyperprolactinaemia.

Physiological	Pathological	Pharmacological
Ovulation Pregnancy Breastfeeding Stress Exercise Nipple stimulation or chest wall injury	Prolactin-secreting pituitary adenoma “Stalk-effect” from sellar/parasellar lesions Renal Failure Liver Cirrhosis Primary hypothyroidism Polycystic Ovarian Syndrome (PCOS) Seizures	Antipsychotics/neuroleptics Antidepressants Antiemetics Opioids Antihypertensives

**Table 2:** Prevalence of hyperprolactinaemia (HPRL) amongst different medications [57,58].

Drug Class	No Significant HPRL	HPRL in <25% of Patients	HPRL in 25–50% of Patients	HPRL in >50% of Patients
Typical antipsychotics		Loxapine Pimozide		Butyrophenone Phenothiazines Thioxanthenes
Atypical antipsychotics	Aripiprazole Clozapine Ziprasidone	Olanzapine Quetiapine		Amisulpride Risperidone Sultopride Sulpiride Tiapride
Tricyclic antidepressants	Nortriptyline	Amitriptyline Amoxapine Clomipramine Desipramine Doxepin Imipramine Maprotiline Trimipramine		Clomipramine
Monoamine oxidase inhibitors				Clorgiline Pargyline
Antiemetics				Alizapride Domperidone Metoclopramide Metopimazine
Antihypertensives		Methyldopa Reserpine	Verapamil	

**Table 3:** Hyperprolactinaemia (HPRL) amongst different antipsychotics.

Drug	Mild HPRL (<50 µg/L) (% of Patients)	Moderate HPRL (50–100 µg/L) (% of Patients)	Severe HPRL (>100 µg/L) (% of Patients)
Aripiprazole *	✓ (0.36)	✓ (2.55)	
Olanzapine *	✓ (26.66)	✓ (11)	✓ (2)
Quetiapine *	✓ (9.09)	✓ (9)	✓ (5)
Depot Risperidone *	✓ (30.76)	✓ (23.07)	✓ (30.79)
Oral Risperidone *	✓ (14.58)	✓ (43.75)	✓ (22.91)
Oral Paliperidone *	✓ (18.18)	✓ (45.45)	✓ (18.18)
Depot Paliperidone *	✓ (13.33)	✓ (40)	✓ (40)
Phenothiazines **			✓
Amisulpride ***			✓ (45%)
Sulpiride ^∞^			✓
Haloperidol ^†^		✓	✓

* [56], ** [60], *** [61], ^∞^ [62], ^†^ [63].

## References

[1] Tucker H.A. (2000). Hormones, mammary growth, and lactation: A 41-year perspective. J. Dairy Sci..

[2] Trott J.F., Vonderhaar B.K., Hovey R.C. (2008). Historical perspectives of prolactin and growth hormone as mammogens, lactogens and galactagogues—Agog for the future!. J. Mammary Gland Biol. Neoplasia.

[3] Riddle O., Bates R.W., Dykshorn S.W. (1933). The preparation, identification and assay of prolactin—A hormone of the anterior pituitary. Am. J. Physiol. Leg. Content.

[4] Friesen H., Guyda H., Hardy J. (1970). Biosynthesis of Human Growth Hormone and Prolactin. J. Clin. Endocrinol. Metab..

[5] Lewis U.J., Singh R.N., Seavey B.K. (1971). Human prolactin: Isolation and some properties. Biochem. Biophys. Res. Commun..

[6] Prabhakar V.K., Davis J.R. (2008). Hyperprolactinaemia. Best Pract. Res. Clin. Obs. Gynaecol..

[7] Teilum K., Hoch J.C., Goffin V., Kinet S., Martial J.A., Kragelund B.B. (2005). Solution structure of human prolactin. J. Mol. Biol..

[8] Freeman M.E., Kanyicska B., Lerant A., Nagy G. (2000). Prolactin: Structure, function, and regulation of secretion. Physiol. Rev..

[9] Capozzi A., Scambia G., Pontecorvi A., Lello S. (2015). Hyperprolactinemia: Pathophysiology and therapeutic approach. Gynecol. Endocrinol..

[10] Rastrelli G., Corona G., Maggi M. (2015). The role of prolactin in andrology: What is new?. Rev. Endocr. Metab. Disord..

[11] Grattan D.R. (2015). 60 YEARS OF NEUROENDOCRINOLOGY: The hypothalamo-prolactin axis. J. Endocrinol..

[12] Cabrera-Reyes E.A., Limón-Morales O., Rivero-Segura N.A., Camacho-Arroyo I., Cerbón M. (2017). Prolactin function and putative expression in the brain. Endocrine.

[13] Marano R.J., Ben-Jonathan N. (2014). Minireview: Extrapituitary Prolactin: An Update on the Distribution, Regulation, and Functions. Mol. Endocrinol..

[14] Ignacak A., Kasztelnik M., Sliwa T., Korbut R.A., Rajda K., Guzik T.J. (2012). Prolactin--not only lactotrophin. A “new” view of the “old” hormone. J. Physiol. Pharm..

[15] Levine S., Muneyyirci-Delale O. (2018). Stress-Induced Hyperprolactinemia: Pathophysiology and Clinical Approach. Obs. Gynecol. Int..

[16] Zaidi M., Sun L., Liu P., Davies T.F., New M., Zallone A., Yuen T. (2016). Pituitary-bone connection in skeletal regulation. Horm. Mol. Biol. Clin. Investig..

[17] Gregg C., Shikar V., Larsen P., Mak G., Chojnacki A., Yong V.W., Weiss S. (2007). White matter plasticity and enhanced remyelination in the maternal CNS. J. Neurosci..

[18] Tanner M.J., Hadlow N.C., Wardrop R. (2011). Variation of female prolactin levels with menopausal status and phase of menstrual cycle. Aust. N. Z. J. Obs. Gynaecol..

[19] Hu Y., Ding Y., Yang M., Xiang Z. (2018). Serum prolactin levels across pregnancy and the establishment of reference intervals. Clin. Chem. Lab. Med..

[20] Alvarez-Tutor E., Forga-LLenas L., Rodriguez-Erdozain R., Goñi-Iriarte M.J., Mendendez-Torre E., Alvarez-Tutor J. (1999). Persistent increase of PRL after oral contraceptive treatment. Alterations in dopaminergic regulation as possible etiology. Arch. Gynecol. Obs..

[21] Banaszewska B., Pawelczyk L., Spaczynski R.Z., Dziura J., Duleba A.J. (2007). Effects of simvastatin and oral contraceptive agent on polycystic ovary syndrome: Prospective, randomized, crossover trial. J. Clin. Endocrinol. Metab..

[22] Crowley W.R. (2015). Neuroendocrine regulation of lactation and milk production. Compr. Physiol..

[23] Muneyyirci-Delale O., Goldstein D., Reyes F.I. (1989). Diagnosis of stress-related hyperprolactinemia. Evaluation of the hyperprolactinemia rest test. N. Y. State J. Med..

[24] Hackney A.C., Davis H.C., Lane A.R. (2016). Growth Hormone-Insulin-Like Growth Factor Axis, Thyroid Axis, Prolactin, and Exercise. Front. Horm. Res..

[25] Melmed S., Casanueva F.F., Hoffman A.R., Kleinberg D.L., Montori V.M., Schlechte J.A., Wass J.A., Society E. (2011). Diagnosis and treatment of hyperprolactinemia: An Endocrine Society clinical practice guideline. J. Clin. Endocrinol. Metab..

[26] Vilar L., Fleseriu M., Bronstein M.D. (2014). Challenges and pitfalls in the diagnosis of hyperprolactinemia. Arq. Bras. Endocrinol. Metab..

[27] Saraç F., Tütüncüoğlu P., Ozgen A.G., Saygili F., Yilmaz C., Bilgen I., Memiş A. (2008). Prolactin levels and examination with breast ultrasound or mammography. Adv. Ther..

[28] Jarrell J., Franks S., McInnes R., Gemayel K., Guyda H., Arronet G.H., Naftolin F. (1980). Breast examination does not elevate serum prolactin. Fertil. Steril..

[29] Hammond K.R., Steinkampf M.P., Boots L.R., Blackwell R.E. (1996). The effect of routine breast examination on serum prolactin levels. Fertil. Steril..

[30] Fernandez A., Karavitaki N., Wass J.A. (2010). Prevalence of pituitary adenomas: A community-based, cross-sectional study in Banbury (Oxfordshire, UK). Clin. Endocrinol. Oxf..

[31] Maiter D., Delgrange E. (2014). Therapy of endocrine disease: The challenges in managing giant prolactinomas. Eur. J. Endocrinol..

[32] Vilar L., Freitas M.C., Naves L.A., Casulari L.A., Azevedo M., Montenegro R., Barros A.I., Faria M., Nascimento G.C., Lima J.G. (2008). Diagnosis and management of hyperprolactinemia: Results of a Brazilian multicenter study with 1234 patients. J. Endocrinol. Investig..

[33] Vilar L., Vilar C.F., Lyra R., Lyra R., Naves L.A. (2017). Acromegaly: Clinical features at diagnosis. Pituitary.

[34] Katznelson L., Laws E.R., Melmed S., Molitch M.E., Murad M.H., Utz A., Wass J.A., Endocrine S. (2014). Acromegaly: An endocrine society clinical practice guideline. J. Clin. Endocrinol. Metab..

[35] Mete O., Lopes M.B. (2017). Overview of the 2017 WHO Classification of Pituitary Tumors. Endocr. Pathol..

[36] Schernthaner-Reiter M.H., Trivellin G., Stratakis C.A. (2016). MEN1, MEN4, and Carney Complex: Pathology and Molecular Genetics. Neuroendocrinology.

[37] Thakker R.V. (2014). Multiple endocrine neoplasia type 1 (MEN1) and type 4 (MEN4). Mol. Cell. Endocrinol..

[38] Agarwal S.K., Ozawa A., Mateo C.M., Marx S.J. (2009). The MEN1 gene and pituitary tumours. Horm. Res..

[39] Beckers A., Aaltonen L.A., Daly A.F., Karhu A. (2013). Familial isolated pituitary adenomas (FIPA) and the pituitary adenoma predisposition due to mutations in the aryl hydrocarbon receptor interacting protein (AIP) gene. Endocr. Rev..

[40] Kurtkaya-Yapicier O., Scheithauer B.W., Carney J.A., Kovacs K., Horvath E., Stratakis C.A., Vidal S., Vella A., Young W.F., Atkinson J.L. (2002). Pituitary adenoma in Carney complex: An immunohistochemical, ultrastructural, and immunoelectron microscopic study. Ultrastruct. Pathol..

[41] Sun F., Sun X., Du X., Xing H., Yang B. (2017). Factors related to endocrine changes and hormone substitution treatment during pre- and post-operation stages in craniopharyngioma. Oncol. Lett..

[42] Karavitaki N., Thanabalasingham G., Shore H.C., Trifanescu R., Ansorge O., Meston N., Turner H.E., Wass J.A. (2006). Do the limits of serum prolactin in disconnection hyperprolactinaemia need re-definition? A study of 226 patients with histologically verified non-functioning pituitary macroadenoma. Clin. Endocrinol. Oxf..

[43] Vilar L., Abucham J., Albuquerque J.L., Araujo L.A., Azevedo M.F., Boguszewski C.L., Casulari L.A., Cunha Neto M.B.C., Czepielewski M.A., Duarte F.H.G. (2018). Controversial issues in the management of hyperprolactinemia and prolactinomas—An overview by the Neuroendocrinology Department of the Brazilian Society of Endocrinology and Metabolism. Arch. Endocrinol. Metab..

[44] Chiloiro S., Giampietro A., Bianchi A., Tartaglione T., Capobianco A., Anile C., De Marinis L. (2017). Diagnosis of endocrine disease: Primary empty sella: A comprehensive review. Eur. J. Endocrinol..

[45] Lo J.C., Beck G.J., Kaysen G.A., Chan C.T., Kliger A.S., Rocco M.V., Chertow G.M., Study F. (2017). Hyperprolactinemia in end-stage renal disease and effects of frequent hemodialysis. Hemodial. Int..

[46] Balakrishnan C.H., Rajeev H. (2017). Correlation of Serum Prolactin Level to Child Pugh Scoring System in Cirrhosis of Liver. J. Clin. Diagn. Res..

[47] Sharma L.K., Sharma N., Gadpayle A.K., Dutta D. (2016). Prevalence and predictors of hyperprolactinemia in subclinical hypothyroidism. Eur. J. Intern. Med..

[48] Honbo K.S., van Herle A.J., Kellett K.A. (1978). Serum prolactin levels in untreated primary hypothyroidism. Am. J. Med..

[49] Shivaprasad K.S., Siddardha K. (2019). Pituitary Hyperplasia from Primary Hypothyroidism. N. Engl. J. Med..

[50] Kyritsi E.M., Dimitriadis G.K., Angelousi A., Mehta H., Shad A., Mytilinaiou M., Kaltsas G., Randeva H.S. (2018). The value of prolactin in predicting prolactinοma in hyperprolactinaemic polycystic ovarian syndrome. Eur. J. Clin. Investig..

[51] Kyritsi E.M., Dimitriadis G.K., Kyrou I., Kaltsas G., Randeva H.S. (2017). PCOS remains a diagnosis of exclusion: A concise review of key endocrinopathies to exclude. Clin. Endocrinol. Oxf..

[52] Filho R.B., Domingues L., Naves L., Ferraz E., Alves A., Casulari L.A. (2007). Polycystic ovary syndrome and hyperprolactinemia are distinct entities. Gynecol. Endocrinol..

[53] Nass R.D., Sassen R., Elger C.E., Surges R. (2017). The role of postictal laboratory blood analyses in the diagnosis and prognosis of seizures. Seizure.

[54] Lusić I., Pintarić I., Hozo I., Boić L., Capkun V. (1999). Serum prolactin levels after seizure and syncopal attacks. Seizure.

[55] Peuskens J., Pani L., Detraux J., De Hert M. (2014). The effects of novel and newly approved antipsychotics on serum prolactin levels: A comprehensive review. CNS Drugs.

[56] Montejo Á., Arango C., Bernardo M., Carrasco J.L., Crespo-Facorro B., Cruz J.J., Del Pino J., García Escudero M.A., García Rizo C., González-Pinto A. (2016). Spanish consensus on the risks and detection of antipsychotic drug-related hyperprolactinaemia. Rev. Psiquiatr. Salud Ment..

[57] Cortet-Rudelli C., Sapin R., Bonneville J.F., Brue T. (2007). Etiological diagnosis of hyperprolactinemia. Ann. Endocrinol. Paris.

[58] Molitch M.E. (2005). Medication-induced hyperprolactinemia. Mayo Clin. Proc..

[59] Ajmal A., Joffe H., Nachtigall L.B. (2014). Psychotropic-induced hyperprolactinemia: A clinical review. Psychosomatics.

[60] Tewksbury A., Olander A. (2016). Management of antipsychotic-induced hyperprolactinemia. Ment. Health Clin..

[61] Reeves S., Sugita E., Baldeweg S.E., Howard R. (2018). Management of antipsychotic related hyperprolactinemia in older people: Can we extrapolate from existing guidance?. Int. J. Geriatr. Psychiatry.

[62] McMurdo M.E., Howie P.W., Lewis M., Marnie M., McEwen J., McNeilly A.S. (1987). Prolactin response to low dose sulpiride. Br. J. Clin. Pharm..

[63] Cookson J., Hodgson R., Wildgust H.J. (2012). Prolactin, hyperprolactinaemia and antipsychotic treatment: A review and lessons for treatment of early psychosis. J. Psychopharmacol..

[64] Ingram J., Taylor H., Churchill C., Pike A., Greenwood R. (2012). Metoclopramide or domperidone for increasing maternal breast milk output: A randomised controlled trial. Arch. Dis. Child. Fetal Neonatal Ed..

[65] Fountas A., Chai S.T., Kourkouti C., Karavitaki N. (2018). Mechanisms of endocrinology: Endocrinology of opioids. Eur. J. Endocrinol..

[66] Romeo J.H., Dombrowski R., Kwak Y.S., Fuehrer S., Aron D.C. (1996). Hyperprolactinaemia and verapamil: Prevalence and potential association with hypogonadism in men. Clin. Endocrinol. Oxf..

[67] Kelley S.R., Kamal T.J., Molitch M.E. (1996). Mechanism of verapamil calcium channel blockade-induced hyperprolactinemia. Am. J. Physiol..

[68] Veselinović T., Schorn H., Vernaleken I.B., Schiffl K., Klomp M., Gründer G. (2011). Impact of different antidopaminergic mechanisms on the dopaminergic control of prolactin secretion. J. Clin. Psychopharmacol..

[69] Kleinberg D.L., Noel G.L., Frantz A.G. (1977). Galactorrhea: A study of 235 cases, including 48 with pituitary tumors. N. Engl. J. Med..

[70] Finken M.J., Boersma B., Rotteveel J. (2013). Hyperprolactinemia and hyperandrogenism in an adolescent girl presenting with primary amenorrhea. Eur. J. Obs. Gynecol. Reprod. Biol..

[71] Majumdar A., Mangal N.S. (2013). Hyperprolactinemia. J. Hum. Reprod. Sci..

[72] Moria Y., Kortbawi R., El-Asmar N., Arafah B.M. (2019). Increased androgen secretion in patients with prolactinomas: The impact of altered HPA function. Pituitary.

[73] Carmina E., Azziz R., Bergfeld W., Escobar Morreale H.F., Futterweit W., Huddleston H., Lobo R.A., Olsen E. (2019). Female pattern hair loss and androgen excess: A report from the multidisciplinary androgen excess and pcos committee. J. Clin. Endocrinol. Metab..

[74] Serri O., Chik C.L., Ur E., Ezzat S. (2003). Diagnosis and management of hyperprolactinemia. Can. Med. Assoc. J..

[75] De Rosa M., Zarrilli S., Di Sarno A., Milano N., Gaccione M., Boggia B., Lombardi G., Colao A. (2003). Hyperprolactinemia in men: Clinical and biochemical features and response to treatment. Endocrine.

[76] Tirosh A., Benbassat C., Lifshitz A., Shimon I. (2015). Hypopituitarism patterns and prevalence among men with macroprolactinomas. Pituitary.

[77] Mazziotti G., Frara S., Giustina A. (2018). Pituitary Diseases and Bone. Endocr. Rev..

[78] Ozer F.F., Dagdelen S., Erbas T. (2018). Relation of RANKL and OPG Levels with Bone Resorption in Patients with Acromegaly and Prolactinoma. Horm. Metab. Res..

[79] Di Somma C., Colao A., Di Sarno A., Klain M., Landi M.L., Facciolli G., Pivonello R., Panza N., Salvatore M., Lombardi G. (1998). Bone marker and bone density responses to dopamine agonist therapy in hyperprolactinemic males. J. Clin. Endocrinol. Metab..

[80] Mazziotti G., Mancini T., Mormando M., De Menis E., Bianchi A., Doga M., Porcelli T., Vescovi P.P., De Marinis L., Giustina A. (2011). High prevalence of radiological vertebral fractures in women with prolactin-secreting pituitary adenomas. Pituitary.

[81] D’Sylva C., Khan T., Van Uum S., Fraser L.A. (2015). Osteoporotic fractures in patients with untreated hyperprolactinemia vs. those taking dopamine agonists: A systematic review and meta-analysis. Neuro Endocrinol. Lett..

[82] Casanueva F.F., Molitch M.E., Schlechte J.A., Abs R., Bonert V., Bronstein M.D., Brue T., Cappabianca P., Colao A., Fahlbusch R. (2006). Guidelines of the Pituitary Society for the diagnosis and management of prolactinomas. Clin. Endocrinol. Oxf..

[83] Saleem M., Martin H., Coates P. (2018). Prolactin Biology and Laboratory Measurement: An Update on Physiology and Current Analytical Issues. Clin. Biochem. Rev..

[84] Lippi G., Plebani M. (2016). Macroprolactin: Searching for a needle in a haystack?. Clin. Chem. Lab. Med..

[85] Samson S.L., Hamrahian A.H., Ezzat S., Aace N., Pituitary Scientific Committee (2015). American association of clinical endocrinologists, American college of endocrinology disease state clinical review: Clinical relevance of macroprolactin in the absence or presence of true hyperprolactinemia. Endocr. Pract..

[86] Kasum M., Orešković S., Čehić E., Šunj M., Lila A., Ejubović E. (2017). Laboratory and clinical significance of macroprolactinemia in women with hyperprolactinemia. Taiwan J. Obs. Gynecol..

[87] Kalsi A.K., Halder A., Jain M., Chaturvedi P.K., Sharma J.B. (2019). Prevalence and reproductive manifestations of macroprolactinemia. Endocrine.

[88] Romijn J.A. (2014). Hyperprolactinemia and prolactinoma. Handb. Clin. Neurol..

[89] Grigg J., Worsley R., Thew C., Gurvich C., Thomas N., Kulkarni J. (2017). Antipsychotic-induced hyperprolactinemia: Synthesis of world-wide guidelines and integrated recommendations for assessment, management and future research. Psychopharmacology.

[90] Santharam S., Fountas A., Tampourlou M., Arlt W., Ayuk J., Gittoes N., Toogood A., Karavitaki N. (2018). Impact of menopause on outcomes in prolactinomas after dopamine agonist treatment withdrawal. Clin. Endocrinol. Oxf..

[91] Iacovazzo D., De Marinis L. (2015). Treatment of hyperprolactinemia in post-menopausal women: Pros. Endocrine.

[92] Liu X., Tang C., Wen G., Zhong C., Yang J., Zhu J., Ma C. (2018). The Mechanism and Pathways of Dopamine and Dopamine Agonists in Prolactinomas. Front. Endocrinol. Lausanne.

[93] Barlier A., Jaquet P. (2006). Quinagolide—A valuable treatment option for hyperprolactinaemia. Eur. J. Endocrinol..

[94] Colao A., Di Sarno A., Guerra E., De Leo M., Mentone A., Lombardi G. (2006). Drug insight: Cabergoline and bromocriptine in the treatment of hyperprolactinemia in men and women. Nat. Clin. Pract. Endocrinol. Metab..

[95] Schade R., Andersohn F., Suissa S., Haverkamp W., Garbe E. (2007). Dopamine agonists and the risk of cardiac-valve regurgitation. N. Engl. J. Med..

[96] Stiles C.E., Tetteh-Wayoe E.T., Bestwick J., Steeds R.P., Drake W.M. (2018). A meta-analysis of the prevalence of cardiac valvulopathy in hyperprolactinemic patients treated with Cabergoline. J. Clin. Endocrinol. Metab..

[97] Auriemma R.S., Pivonello R., Ferreri L., Priscitelli P., Colao A. (2015). Cabergoline use for pituitary tumors and valvular disorders. Endocrinol. Metab. Clin. N. Am..

[98] Vroonen L., Lancellotti P., Garcia M.T., Dulgheru R., Rubio-Almanza M., Maiga I., Magne J., Petrossians P., Auriemma R., Daly A.F. (2017). Prospective, long-term study of the effect of cabergoline on valvular status in patients with prolactinoma and idiopathic hyperprolactinemia. Endocrine.

[99] Gamble D., Fairley R., Harvey R., Farman C., Cantley N., Leslie S.J. (2017). Screening for valve disease in patients with hyperprolactinaemia disorders prescribed cabergoline: A service evaluation and literature review. Adv. Drug Saf..

[100] Steeds R.P., Stiles C.E., Sharma V., Chambers J.B., Lloyd G., Drake W. (2019). Echocardiography and monitoring patients receiving dopamine agonist therapy for hyperprolactinaemia: A joint position statement of the British Society of Echocardiography, the British Heart Valve Society and the Society for Endocrinology. Echo Res. Pract..

[101] Gillam M.P., Molitch M.E., Lombardi G., Colao A. (2006). Advances in the treatment of prolactinomas. Endocr. Rev..

[102] Noronha S., Stokes V., Karavitaki N., Grossman A. (2016). Treating prolactinomas with dopamine agonists: Always worth the gamble?. Endocrine.

[103] Chng E., Dalan R. (2013). Pituitary apoplexy associated with cabergoline therapy. J. Clin. Neurosci..

[104] Ghadirian H., Shirani M., Ghazi-Mirsaeed S., Mohebi S., Alimohamadi M. (2018). Pituitary Apoplexy during Treatment of Prolactinoma with Cabergoline. Asian J. Neurosurg..

[105] Glezer A., Bronstein M.D. (2015). Pituitary apoplexy: Pathophysiology, diagnosis and management. Arch. Endocrinol. Metab..

[106] Huang W., Molitch M.E. (2019). Pituitary Tumors in Pregnancy. Endocrinol. Metab. Clin. N. Am..

[107] Auriemma R.S., Perone Y., Di Sarno A., Grasso L.F., Guerra E., Gasperi M., Pivonello R., Colao A. (2013). Results of a single-center observational 10-year survey study on recurrence of hyperprolactinemia after pregnancy and lactation. J. Clin. Endocrinol. Metab..

[108] Domingue M.E., Devuyst F., Alexopoulou O., Corvilain B., Maiter D. (2014). Outcome of prolactinoma after pregnancy and lactation: A study on 73 patients. Clin. Endocrinol. Oxf..

[109] Souteiro P., Karavitaki N. (2019). Dopamine agonist resistant prolactinomas: Any alternative medical treatment?. Pituitary.

[110] Maiter D. (2019). Management of Dopamine Agonist-Resistant Prolactinoma. Neuroendocrinology.

[111] Molitch M.E. (2014). Management of medically refractory prolactinoma. J. Neurooncol..

[112] Raverot G., Burman P., McCormack A.I., Heaney A.P., Petersenn S., Popovic V., Trouillas J., Dekkers O. (2017). European Society of Endocrinology clinical practice guidelines for the management of aggressive pituitary tumours and carcinomas. Eur. J. Endocrinol..

[113] Nakhleh A., Shehadeh N., Hochberg I., Zloczower M., Zolotov S., Taher R., Daoud Naccache D. (2018). Management of cystic prolactinomas: A review. Pituitary.

[114] Tampourlou M., Trifanescu R., Paluzzi A., Ahmed S.K., Karavitaki N. (2016). Therapy of endocrine disease: Surgery in microprolactinomas: Effectiveness and risks based on contemporary literature. Eur. J. Endocrinol..

[115] Colao A. (2009). Pituitary tumours: The prolactinoma. Best Pract. Res. Clin. Endocrinol. Metab..

[116] Han Y.L., Chen D.M., Zhang C., Pan M., Yang X.P., Wu Y.G. (2018). Retrospective analysis of 52 patients with prolactinomas following endoscopic endonasal transsphenoidal surgery. Med. Baltim..

[117] Ntali G., Karavitaki N. (2015). Efficacy and complications of pituitary irradiation. Endocrinol. Metab. Clin. N. Am..

[118] Pereira A.M., Romijn J.A., Dekkers O.M. (2008). Treatment and Follow-Up of Clinically Nonfunctioning Pituitary Macroadenomas. J. Clin. Endocrinol. Metab..

[119] Zaidi H.A., Cote D.J., Castlen J.P., Burke W.T., Liu Y.H., Smith T.R., Laws E.R. (2017). Time Course of Resolution of Hyperprolactinemia After Transsphenoidal Surgery Among Patients Presenting with Pituitary Stalk Compression. World Neurosurg..

[120] Huang W., Molitch M.E. (2012). Evaluation and management of galactorrhea. Am. Fam. Physician.

[121] Greenman Y., Cooper O., Yaish I., Robenshtok E., Sagiv N., Jonas-Kimchi T., Yuan X., Gertych A., Shimon I., Ram Z. (2016). Treatment of clinically nonfunctioning pituitary adenomas with dopamine agonists. Eur. J. Endocrinol..

[122] Ben-Jonathan N., Hugo E. (2015). Prolactin (PRL) in adipose tissue: Regulation and functions. Adv. Exp. Med. Biol..

[123] Carré N., Binart N. (2014). Prolactin and adipose tissue. Biochimie.

[124] Wang T., Lu J., Xu Y., Li M., Sun J., Zhang J., Xu B., Xu M., Chen Y., Bi Y. (2013). Circulating prolactin associates with diabetes and impaired glucose regulation: A population-based study. Diabetes Care.

[125] Li J., Rice M.S., Huang T., Hankinson S.E., Clevenger C.V., Hu F.B., Tworoger S.S. (2018). Circulating prolactin concentrations and risk of type 2 diabetes in US women. Diabetologia.

[126] Atmaca A., Bilgici B., Ecemis G.C., Tuncel O.K. (2013). Evaluation of body weight, insulin resistance, leptin and adiponectin levels in premenopausal women with hyperprolactinemia. Endocrine.

[127] Dos Santos Silva C.M., Barbosa F.R., Lima G.A., Warszawski L., Fontes R., Domingues R.C., Gadelha M.R. (2011). BMI and metabolic profile in patients with prolactinoma before and after treatment with dopamine agonists. Obes. Silver Spring.

[128] Schwetz V., Librizzi R., Trummer C., Theiler G., Stiegler C., Pieber T.R., Obermayer-Pietsch B., Pilz S. (2017). Treatment of hyperprolactinaemia reduces total cholesterol and LDL in patients with prolactinomas. Metab. Brain Dis..

[129] Medic-Stojanoska M., Icin T., Pletikosic I., Bajkin I., Novakovic-Paro J., Stokic E., Spasic D.T., Kovacev-Zavisic B., Abenavoli L. (2015). Risk factors for accelerated atherosclerosis in young women with hyperprolactinemia. Med. Hypotheses.

[130] Auriemma R.S., Granieri L., Galdiero M., Simeoli C., Perone Y., Vitale P., Pivonello C., Negri M., Mannarino T., Giordano C. (2013). Effect of cabergoline on metabolism in prolactinomas. Neuroendocrinology.

[131] Auriemma R.S., Galdiero M., Vitale P., Granieri L., Lo Calzo F., Salzano C., Ferreri L., Pivonello C., Cariati F., Coppola G. (2015). Effect of chronic cabergoline treatment and testosterone replacement on metabolism in male patients with prolactinomas. Neuroendocrinology.

[132] Ozdemir E.D., Caglar G.S., Akgul E., Cengiz S.D., Tombak G. (2014). The association between prolactin, high-sensitivity C-reactive protein and Framingham risk score in menopause. Gynecol. Obs. Investig..

[133] Fleseriu M., Hashim I.A., Karavitaki N., Melmed S., Murad M.H., Salvatori R., Samuels M.H. (2016). Hormonal Replacement in Hypopituitarism in Adults: An Endocrine Society Clinical Practice Guideline. J. Clin. Endocrinol. Metab..

[134] Zhang L., Curhan G.C., Forman J.P. (2010). Plasma prolactin level and risk of incident hypertension in postmenopausal women. J. Hypertens..

[135] Therkelsen K.E., Abraham T.M., Pedley A., Massaro J.M., Sutherland P., Hoffmann U., Fox C.S. (2016). Association Between Prolactin and Incidence of Cardiovascular Risk Factors in the Framingham Heart Study. J. Am. Heart Assoc..

[136] Erem C., Kocak M., Nuhoglu I., Yılmaz M., Ucuncu O. (2010). Blood coagulation, fibrinolysis and lipid profile in patients with prolactinoma. Clin. Endocrinol. Oxf..

[137] Toulis K.A., Robbins T., Reddy N., Balachandran K., Gokhale K., Wijesinghe H., Cheng K.K., Karavitaki N., Wass J., Nirantharakumar K. (2018). Males with prolactinoma are at increased risk of incident cardiovascular disease. Clin. Endocrinol. Oxf..

[138] Shelly S., Boaz M., Orbach H. (2012). Prolactin and autoimmunity. Autoimmun. Rev..

[139] Orbach H., Zandman-Goddard G., Boaz M., Agmon-Levin N., Amital H., Szekanecz Z., Szucs G., Rovensky J., Kiss E., Doria A. (2012). Prolactin and autoimmunity: Hyperprolactinemia correlates with serositis and anemia in SLE patients. Clin. Rev. Allergy Immunol..

[140] Correale J., Farez M.F., Ysrraelit M.C. (2014). Role of prolactin in B cell regulation in multiple sclerosis. J. Neuroimmunol..

[141] Liu Y., Zhang Z., Jin Q., Kang Z., Huo Y., He Z., Feng X., Yin J., Wu X., Wang H. (2019). Hyperprolactinemia is associated with a high prevalence of serum autoantibodies, high levels of inflammatory cytokines and an abnormal distribution of peripheral B-cell subsets. Endocrine.

[142] Wang P., Lv T.T., Guan S.Y., Li H.M., Leng R.X., Zou Y.F., Pan H.F. (2017). Increased plasma/serum levels of prolactin in systemic lupus erythematosus: A systematic review and meta-analysis. Postgrad. Med..

[143] Aulestia C., De Zubiría A., Granados C., Suárez J., Cervera R. (2016). Prolactin and Estradiol Profile in a Cohort of Colombian Women with Systemic Lupus Erythematosus. Isr. Med. Assoc. J..

[144] Ledesma-Soto Y., Blanco-Favela F., Fuentes-Pananá E.M., Tesoro-Cruz E., Hernández-González R., Arriaga-Pizano L., Legorreta-Haquet M.V., Montoya-Diaz E., Chávez-Sánchez L., Castro-Mussot M.E. (2012). Increased levels of prolactin receptor expression correlate with the early onset of lupus symptoms and increased numbers of transitional-1 B cells after prolactin treatment. BMC Immunol..

[145] Savino W. (2017). Prolactin: An Immunomodulator in Health and Disease. Front. Horm. Res..

[146] Leaños-Miranda A., Cárdenas-Mondragón G. (2006). Serum free prolactin concentrations in patients with systemic lupus erythematosus are associated with lupus activity. Rheumatol. Oxf..

[147] Tang M.W., Garcia S., Gerlag D.M., Tak P.P., Reedquist K.A. (2017). Insight into the Endocrine System and the Immune System: A Review of the Inflammatory Role of Prolactin in Rheumatoid Arthritis and Psoriatic Arthritis. Front. Immunol..

[148] Clapp C., Adán N., Ledesma-Colunga M.G., Solís-Gutiérrez M., Triebel J., Martínez de la Escalera G. (2016). The role of the prolactin/vasoinhibin axis in rheumatoid arthritis: An integrative overview. Cell. Mol. Life Sci..

[149] Borba V.V., Zandman-Goddard G., Shoenfeld Y. (2018). Prolactin and Autoimmunity. Front. Immunol..

[150] Salesi M., Sadeghihaddadzavareh S., Nasri P., Namdarigharaghani N., Farajzadegan Z., Hajalikhani M. (2013). The role of bromocriptine in the treatment of patients with active rheumatoid arthritis. Int. J. Rheum. Dis..

[151] Tang M.W., Garcia S., Malvar Fernandez B., Gerlag D.M., Tak P.P., Reedquist K.A. (2017). Rheumatoid arthritis and psoriatic arthritis synovial fluids stimulate prolactin production by macrophages. J. Leukoc. Biol..

[152] Reyes-Castillo Z., Pereira-Suárez A.L., Palafox-Sanchez C.A., Rangel-Villalobos H., Estrada-Chávez C., Oregón-Romero E., Angel-Chávez L.I., Muñoz-Barrios S., Bueno-Topete M.R., Muñoz-Valle J.F. (2013). The extrapituitary prolactin promoter polymorphism is associated with rheumatoid arthritis and anti-CCP antibodies in Mexican population. Gene.

[153] Türkoğlu R., Giriş M., Gencer M., Akcan U., Örçen A. (2016). Serum Prolactin Levels in Multiple Sclerosis, Neuromyelitis Optica, and Clinically Isolated Syndrome Patients. Noro Psikiyatr. Arsivi.

[154] El Tahlawi S.M., El Eishi N.H., Kahhal R.K., Hegazy R.A., El Hanafy G.M., Abdel Hay R.M., Shaker O.G. (2018). Do Prolactin and its Receptor Play a Role in Alopecia Areata?. Indian J. Derm..

[155] Lajevardi V., Hallaji Z., Daneshpazhooh M., Ghandi N., Shekari P., Khani S. (2016). Evaluation of prolactin levels in patients with newly diagnosed pemphigus vulgaris and its correlation with pemphigus disease area index. Int. J. Womens Derm..

[156] Tworoger S.S., Hankinson S.E. (2008). Prolactin and breast cancer etiology: An epidemiologic perspective. J. Mammary Gland Biol. Neoplasia.

[157] Tworoger S.S., Eliassen A.H., Zhang X., Qian J., Sluss P.M., Rosner B.A., Hankinson S.E. (2013). A 20-year prospective study of plasma prolactin as a risk marker of breast cancer development. Cancer Res..

[158] Bernard V., Young J., Chanson P., Binart N. (2015). New insights in prolactin: Pathological implications. Nat. Rev. Endocrinol..

[159] Clendenen T.V., Arslan A.A., Lokshin A.E., Liu M., Lundin E., Koenig K.L., Berrino F., Hallmans G., Idahl A., Krogh V. (2013). Circulating prolactin levels and risk of epithelial ovarian cancer. Cancer Causes Control.

[160] Ilan Y., Sibirsky O., Livni N., Gofrit O., Barack V., Goldin E. (1995). Plasma and tumor prolactin in colorectal cancer patients. Dig. Dis. Sci..

[161] Bhatavdekar J.M., Patel D.D., Giri D.D., Karelia N.H., Vora H.H., Ghosh N., Shah N.G., Trivedi S.N., Balar D.B. (1992). Comparison of plasma prolactin and CEA in monitoring patients with adenocarcinoma of colon and rectum. Br. J. Cancer.

[162] Yeh Y.T., Lee K.T., Tsai C.J., Chen Y.J., Wang S.N. (2012). Prolactin promotes hepatocellular carcinoma through Janus kinase 2. World J. Surg..

[163] Yonezawa T., Chen K.H., Ghosh M.K., Rivera L., Dill R., Ma L., Villa P.A., Kawaminami M., Walker A.M. (2015). Anti-metastatic outcome of isoform-specific prolactin receptor targeting in breast cancer. Cancer Lett..

[164] Goffin V. (2017). Prolactin receptor targeting in breast and prostate cancers: New insights into an old challenge. Pharm. Ther..

[165] Ding K., Yuan Y., Chong Q.Y., Yang Y., Li R., Li X., Kong X., Qian P., Xiong Z., Pandey V. (2017). Autocrine Prolactin Stimulates Endometrial Carcinoma Growth and Metastasis and Reduces Sensitivity to Chemotherapy. Endocrinology.

[166] Neradugomma N.K., Subramaniam D., Tawfik O.W., Goffin V., Kumar T.R., Jensen R.A., Anant S. (2014). Prolactin signaling enhances colon cancer stemness by modulating Notch signaling in a Jak2-STAT3/ERK manner. Carcinogenesis.

[167] Dekkers O.M., Romijn J.A., de Boer A., Vandenbroucke J.P. (2010). The risk for breast cancer is not evidently increased in women with hyperprolactinemia. Pituitary.

